# An initial framework for use of ultrasound by speech and language therapists in the UK: Scope of practice, education and governance

**DOI:** 10.1177/1742271X221122562

**Published:** 2022-10-12

**Authors:** Jodi Elizabeth Allen, Joanne Cleland, Mike Smith

**Affiliations:** 1 The National Hospital for Neurology and Neurosurgery, University College London NHS Foundation Trust, London, UK; 2 School of Psychological Sciences and Health, University of Strathclyde, Glasgow, UK; 3 School of Healthcare Sciences, Cardiff University, Cardiff, UK

**Keywords:** Speech and language therapy, sonography, speech, voice, swallowing

## Abstract

**Background:**

There is growing evidence to support the use of ultrasound as a tool for the assessment and treatment of speech, voice and swallowing disorders across the Speech and Language Therapy profession. Research has shown that development of training competencies, engagement with employers and the professional body are vital to progressing ultrasound into practice.

**Methods:**

We present a framework to support translation of ultrasound into Speech and Language Therapy. The framework comprises three elements: (1) scope of practice, (2) education and competency and (3) governance. These elements align to provide a foundation for sustainable and high-quality ultrasound application across the profession.

**Results:**

Scope of practice includes the tissues to be imaged, the clinical and sonographic differentials and subsequent clinical decision-making. Defining this provides transformational clarity to Speech and Language Therapists, other imaging professionals and those designing care pathways. Education and competency are explicitly aligned with the scope of practice and include requisite training content and mechanisms for supervision/support from an appropriately trained individual in this area. Governance elements include legal, professional and insurance considerations. Quality assurance recommendations include data protection, storage of images, testing of ultrasound devices as well as continuous professional development and access to a second opinion.

**Conclusion:**

The framework provides an adaptable model for supporting expansion of ultrasound across a range of Speech and Language Therapy specialities. By taking an integrated approach, this multifaceted solution provides the foundation for those with speech, voice and swallowing disorders to benefit from advances in imaging-informed healthcare.

## Introduction

The use of ultrasound (US) imaging outside of traditional radiology settings is an area of rapid growth. In the Speech and Language Therapy (SLT) profession, there is growing evidence to support its application as a tool for the assessment and treatment of speech and swallowing disorders.^1–4^

There are fundamental constraints to integration of US into SLT clinical practice such as cost, availability of equipment and lack of focused training.^2,5^ There is, however, a drive among the profession to start adopting US into practice while research to establish reliability of data acquisition and interpretation continues. To do this, mechanisms to address concerns around competency and scope of practice are required.

US imaging is a modality that requires experience to use and interpret. The skill and experience required by Speech and Language Therapists (SLTs) will vary according to the purpose for which US is being used. The paper uses a framework to describe the scope of practice, education and governance requirements associated with application of US in the profession. This has been used in other allied health professional (AHP) groups in the emerging areas of lung^6^ and pelvic health^7^ to address concerns such as quality assurance and professional indemnity. This paper therefore shares some generic content with these publications,^6,7^ which in turn overlaps with relevant professional guidance.^8,9^

## Speech & language therapists

In the United Kingdom, SLTs are autonomous clinicians who hold a formal qualification as a speech and language therapist. Typically, this will be a minimum of a BSc (Hons) Clinical Communication Studies/Speech Pathology and Therapy or post-graduate equivalent (e.g. MMedSci Clinical Communication Studies). Combined with their professional registration with the Health and Care Professions Council (HCPC), they can use the protected title of ‘Speech and Language Therapist’ and are eligible to join the professional body ‘The Royal College of Speech and Language Therapists (RCSLT)’.

SLTs work across a range of patient and client groups across the lifespan (*the term patient will henceforth refer to both patients and clients*). Their key responsibilities include the assessment, management and monitoring of swallowing and communication, including speech disorders. Assessment includes clinical history-taking alongside a combination of assessment procedures. For swallowing, assessments may include a clinical bedside assessment or instrumental assessment via videofluoroscopic swallowing study (VFSS) or flexible endoscopic evaluation of swallowing (FEES), whereas assessment of both speech and voice quality may include perceptual, acoustic, and instrumental analysis, for example, electropalatography^10^ and vocal acoustic analysis.^11^ Applying a combination of clinical reasoning and patient-centred care, SLTs independently formulate and apply treatment approaches such as exercises to target the underlying speech, voice, or swallowing disorder and/or strategies to reduce the functional impact of the impairment.

SLTs work closely with other professionals such as radiologists, audiologists, ear, nose and throat specialists, intensivists, neurologists, respiratory physicians and physiotherapists to enable interdisciplinary assessment and management of speech, voice and swallowing disorders. In this regard, there may be a degree of overlap with, and aspects of this framework may apply to, other professional groups.

## Applications of US across SLT

There are several possible applications of US across the SLT profession. Some applications, such as use of US for biofeedback in speech disorder intervention, are an already established part of clinical practice in some areas of the United Kingdom.^12^ Other applications, such as its use as a tool to assess dysphagia, remain solely in the research setting. Current and potential application of US across the profession, plus research evidence, have previously been described^13,14^ and are summarised according to SLT role across the full range of patient clinical presentations in Table 1.

**Table 1.** Aims and role of speech and language therapy for speech, voice and swallowing presentations, including ultrasound role.

**Clinical presentation**	Aims and role of speech and language therapy, grouped according to (1) assessment and diagnosis, (2) treatment and outcome measurement (3) integration with wider MDT (current/potential role for ultrasound imaging in **bold**)
Oro-pharyngeal dysphagia	(1) **Screening** for presence versus absence of oro-pharyngeal dysphagia Differentiate actual or likely aetiology of oro-pharyngeal dysphagia (more specifically, **skill** versus **strength**-based impairment via assessment of swallowing **muscle size, structure, and kinematics**) as a foundation for subsequent management Assessment of swallowing **symptoms (aspiration and residue)** (2) Informed by the above, treatment approaches include **education**, **skill-training (using biofeedback)** and strength training **Measuring outcomes** of skill or strength-based treatment which includes measurement of swallowing symptoms (aspiration and residue), changes in **swallowing kinematics** and changes in **muscle size and structure** (3) **Communication of findings** and management approach to patient and other care pathway members. Where appropriate, liaison with other healthcare team members for further investigation and intervention
Dysphonia (and other diagnosis^a^ caused by an impairment of vocal fold movement)	(1) Differentiate actual or likely aetiology^b^ of dysphonia (more specifically, structural, or **kinematic impairment** of vocal fold mobility) as a foundation for subsequent management (2) Informed by the above, treatment approaches include **education**, environmental and behavioural adaptation (such as postural adjustment), surgical intervention (such as vocal fold augmentation) and **exercise-based therapy (including biofeedback)** **Measuring outcomes** of treatment interventions which includes measurement of vocal fold movement. (3) **Communication of findings** and management approach to patient and other care pathway members. Where appropriate, liaison with other healthcare team members for further investigation and intervention
Developmental speech sound disorders	(1) Differentiate actual or likely aetiology of speech sound disorder (more specifically, phonological, or articulatory/motor **impairment of tongue shape, placement, and kinematics**) as a foundation for subsequent management (2) Informed by the above, treatment approaches include **biofeedback** **Measuring outcomes** of treatment interventions which include measurement of **tongue shape and kinematics**. (3) **Communication of findings** and management approach to patient and other care pathway members. Where appropriate, liaison with other healthcare team members for further investigation and intervention
Cleft lip and palate	(1) Differentiate actual or likely aetiology of compensatory articulations (more specifically, **impairment of tongue shape, placement, and kinematics**) as a foundation for subsequent management (2) Informed by the above, treatment approaches include **biofeedback.** **Measuring outcomes** of treatment interventions which include measurement of **tongue shape, placement and kinematics** (3) **Communication of findings** and management approach to patient and other care pathway members. Where appropriate, liaison with other healthcare team members for further investigation and intervention
Acquired speech disorders associated with neurological insult/injury (i.e. apraxia of speech, dysarthria) or surgery/radiation to the structures associated with speech articulation (such as glossectomy)	(4) Differentiate actual or likely aetiology of speech disorder (more specifically, dysarthria or apraxia or **impairment of tongue shape, placement and kinematics**) as a foundation for subsequent management (5) Informed by the above, treatment approaches include **biofeedback** **Measuring outcomes** of treatment interventions which includes measurement of **tongue shape and kinematics** (6) **Communication of findings** and management approach to patient and other care pathway members. Where appropriate, liaison with other healthcare team members for further investigation and intervention
Injection of botulinum toxin (botox) into the salivary glands	(1) **Anatomical location of the (parotid, submandibular & sub-lingual) salivary glands** **Confirmation of injection location for botulinum toxin into the salivary glands**

a For example, stridor, laryngospasm, inducible laryngeal obstruction (ILO).

b In conjunction with ENT (and/or consultant respiratory physician for diagnosis of ILO).

In order to define SLT scope of practice, education and governance, the applications described in Table 1 have been categorised into three domains. These are

1. Static imaging of speech, voice, and swallowing structures
2. Qualitative evaluation of speech, voice and swallowing movement
3. Quantitative analysis of speech, voice and swallowing movement

The three domains are described in Table 2.

**Table 2.** Applications of ultrasound across speech and language therapy divided into three domains: (1) static imaging of speech, voice and swallowing structures; (2) qualitative evaluation of speech, voice and swallowing movement; (3) quantitative analysis of speech, voice, and swallowing movement.

Domain	Purpose	Example	Useful references	Patient group	Alternative approach(es)^a^
**Static imaging** of structures involved in speech, voice & swallowing	Professional training	Pre-registration SLTs to support knowledge acquisition of speech, voice and swallowing anatomy	(1,2)	Not applicable	X-ray, computed tomography (CT), magnetic resonance imaging (MRI) Medical illustrations Cadaver
		Post-registration SLTs to support knowledge acquisition of head and neck imaging modalities and/or as a pre-cursor for use as a speech and swallowing assessment tool	As above	Not applicable	X-ray, computerised tomography (CT), magnetic resonance imaging (MRI) Medical illustrations Cadaver
	Patient education	To educate patients in the anatomy associated with normal speech, voice, and swallowing	As above	Any patients able to participate in US assessment & be supported to understand the findings	Medical illustrations 3D models Video clips/education Apps
		To educate patients in the altered anatomy affecting speech, voice and swallowing associated with their condition	As above	May include (but not limited to) patients with head and neck cancer pre- and post-surgical resection or patients with cleft lip and palate	Medical illustrations 3D models Video clips/education Apps
	Clinical assessment^b^ (+/– outcome measurement)	Detection of bolus* material in the pharynx or larynx to identify the symptoms (aspiration or residues) of swallowing disorder	(3–5)	Patients with dysphagia thought to cause residue or aspiration	Flexible endoscopic evaluation of swallowing (FEES) Videofluoroscopic evaluation of swallowing (VFSS)
		Assessment of the upper and large airway for a range of possible functions, for example, identifying subglottic stenosis, tracheomalacia or predicting endotracheal or tracheostomy size	(6,7)	Patients requiring elective or urgent airway assessment	Endoscopic evaluation of the larynx (EEL) Bronchoscopy MRI CT Microlaryngoscopy
		Measurement of the size and echogenicity of the muscles involved in swallowing to determine atrophy and fat infiltration	(8–10)	Patients with a disease known (or suspected) to cause muscle wasting/atrophy (e.g. motor neuron disease) Patients who have undergone treatments known to cause structural changes in muscle fibres (e.g. radiotherapy) Patients who have not used their speech/swallowing muscles for a period time and have anticipated muscle changes associated with disuse	Magnetic resonance imaging (MR)
	Clinical treatment	Detection of salivary glands for the purpose of botulin toxin injection	(11)	Patients with sialorrhea	Anatomical palpation
**Qualitative evaluation of movement** involved in speech, voice and swallowing	Clinical treatment	Treatment of speech sound disorders, including cleft lip and palate	(12–15)	Patients with hearing impairment, Down’s syndrome, cleft lip and palate, childhood apraxia of speech, childhood dysarthria, and persistent or residual speech sound disorder of unknown origin	Perception-based interventions Electropalatography Acoustic biofeedback Electromagnetic articulography
		Treatment of swallowing disorders	(16)	Patients undergoing dysphagia therapy which target movements visible on US	Surface electromyography (sEMG) Anatomical palpation
	Clinical Assessment (+/– outcome measurement)	Assessment of vocal fold adduction and abduction to assess, for example, presence/absence of vocal fold palsy, paradoxical vocal fold movements/inducible laryngeal obstruction, airway protection for swallowing	(17–19)	Patients with suspected impairment of vocal fold mobility	Perceptual assessment Fibreoptic nasendoscopic examination (FNE) Videolaryngoscopy CT
		Assessment of tongue kinematics	(20,21)	Patients with diagnosis known to cause disorders of tongue movement related to speech or swallowing, for example, those with hearing impairment, craniofacial abnormalities, tumour, apraxia	Electropalatography Electromagnetic articulography
**Quantitative analysis of movement** involved in speech, voice and swallowing	Clinical Assessment (+/– outcome measurement)	A screening tool to determine the presence or absence of swallowing disorder	(22,23)		Various including timed water swallow test and patient-reported tools
		An assessment tool to determine the severity of specific parameters of movement associated with speech, voice or swallowing disorder	(24)	Patients with a diagnosis known to cause disorders of speech, voice or swallowing.	VFSS FEES FNE Electropalatography Acoustic or aerodynamic analysis

a Under IR(MR)R 2019, only non-medical referrers that are suitably state registered are permitted to request imaging tests that involve radiation.

b While image *analysis* for clinical assessment is likely to be static for bolus residue and the upper airway, image *acquisition* may be dynamic (e.g. bolus residue may be assessed over a specified time). For this reason, clinical assessment within this domain may be considered under the categories of either qualitative or quantitative analysis of movement, depending on the exact purpose of the examination. *Bolus refers to food and drink material that has been swallowed.

## A framework approach to supporting use of US in SLT

**Figure 1.**
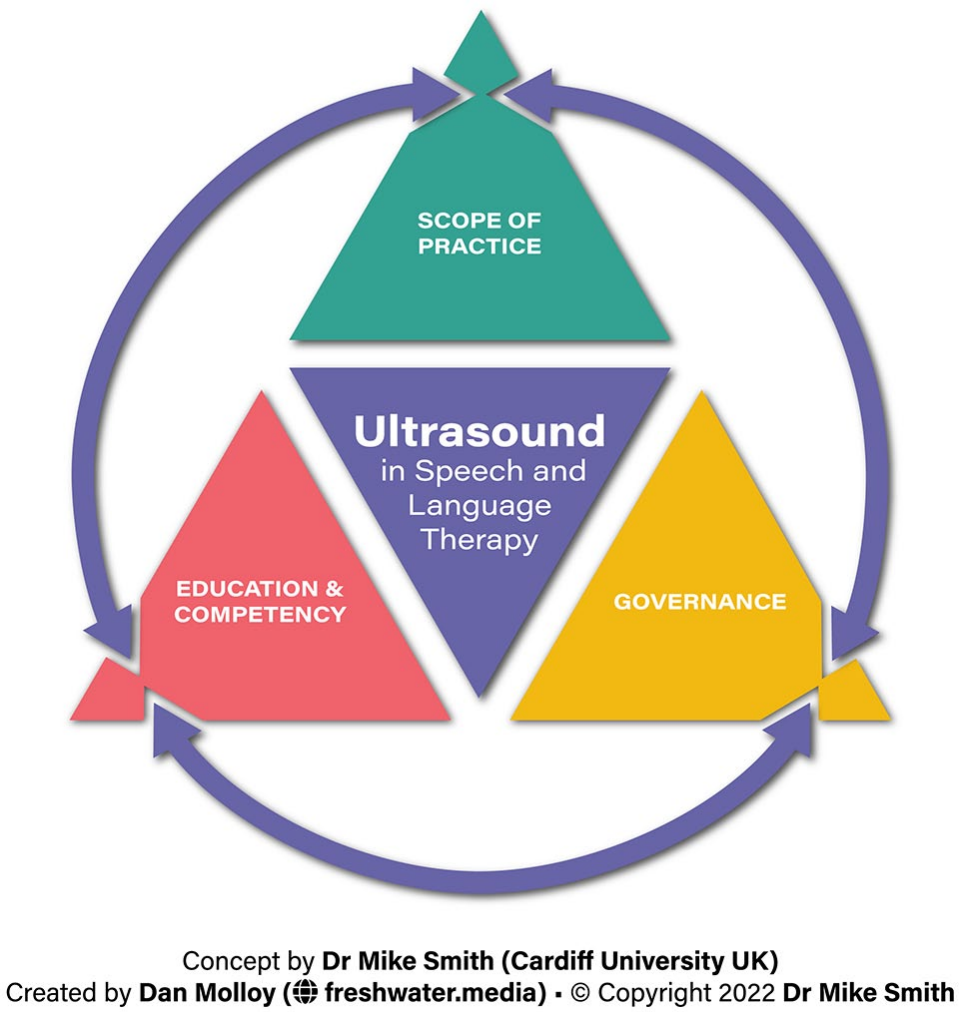
A framework approach to supporting use of US in SLT comprising the elements of (1) scope of practice, (2) education and competency and (3) governance.

The movement towards use of US as an SLT assessment or diagnostic tool necessitates the need for quality assurance and clarity of the SLT role. Recognising this, we propose the use of a framework to support application of US in SLT (Figure 1), comprising the elements of (1) scope of practice, (2) education and competency and (3) governance for each of the uses of US in the SLT profession. The framework uses each element to ensure robust delivery of US across the profession. The same approach has been utilised by other professional groups^6,7^ and therefore ensures application of US in SLT is consistent with other AHP groups. In the same way, new areas of US activity can be established by developing or revising one or more of the elements, thereby ensuring alignment across the framework.

## Scope of practice: clinical and sonographic

Scope of practice refers to numerous elements, including the tissues to be imaged, the clinical and sonographic differentials, subsequent clinical decision-making and reporting.

As the uses of US in SLT are at different stages of development, scope of practice will depend on the specific area in which the SLT wishes to practice and the multidisciplinary support available to them. The combined (1) clinical usability and (2) clinical utility of US imaging according to the literature are key determinants of the scope of practice in this area.

Table 3 provides an indicative list of imaging that may be performed according to the domains described in Table 2. How the device is used depends on the purpose for which US has been selected by SLT clinicians. Emphasis is currently upon static imaging of structures involved in speech, voice and swallowing as part of staff and patient education as well as qualitative evaluation of structures involved in speech, voice and swallowing for the purpose of biofeedback therapy.

**Table 3.** Indicative imaging performed and how this information is used by US SLT clinicians.

Domain	Indicative imaging performed	Role of the imaging of these structures	Role of SLT clinicians in context of patient management
**Static imaging** of structures involved in speech, voice & swallowing	Identification of normal anatomy associated with speech and swallowing Bones/cartilages^a^: • Hyoid • Thyroid • Cricoid • Epiglottis • Arytenoid (left & right) • Hard palate • Tracheal rings Soft tissue structures/muscle: • Tongue (genioglossus) • Soft palate • Geniohyoid • Anterior belly digastric • Mylohyoid • False vocal folds (left & right) • True vocal folds (left & right) • Salivary glands • Masseter • Upper oesophageal sphincter **Further imaging** (as appropriate to role and emerging research evidence) Bolus residue/presence within head/neck structures to include • Pyriform fossae (left & right) • Valleculae (left & right) • Laryngeal vestibule • Tracheal rings (subglottis) Measurements of muscle size & echogenicity to include: • Genioglossus • Masseter • Anterior belly digastric • Geniohyoid	• Awareness of spectrum of ‘normal’ presentations • Landmark identification serves as mechanism to enhance accuracy of imaging; integral aspect of protocol-based imaging	Recognition of ‘normal’ as part of sonographic and clinical differential diagnosis process Standardised approach to imaging as quality assurance mechanism
**Qualitative evaluation of movement** involved in speech, voice and swallowing	Identification of ultrasound appearances of normal position and gross (normal) dynamics • Binary vocal fold movement (unilateral & bilateral) • Tongue movement in swallowing • Tongue movement for speech sounds • Presence/absence of hyoid movement **Further imaging** (as appropriate to role and emerging research evidence) • Disorders of timing	• Awareness of ‘normal’ movement for speech, voice and swallow • Gross differential between normal and disordered movements which include (e.g. glossopalatal seal during bolus hold, bilateral vs unilateral vocal fold movement, fronting/backing/distortion of speech sounds, sustained versus non-sustained hyoid movement) • Biofeedback/therapy function to support therapy from disordered to normal function. • Dysphagia screening tool	Recognition of ‘normal’ versus abnormal speech and swallowing movement as part of sonographic and clinical differential diagnosis process To serve as a therapy/biofeedback tool for gross disorders of swallowing or speech
**Quantitative analysis of movement** involved in speech, voice and swallowing	Identification of ultrasound appearances of normal position and refined dynamics • Grades of vocal fold movement • Grades of tongue movement • Grades of hyoid movement **Further imaging** may evolve as research evidence emerges.	Allows for refined sonographic identification of non-normal/pathological presentations	Initially as an educational mechanism for both SLT staff, MDT & patient (as appropriate) Provides foundation for exploration of US as a potential assessment adjunct (alongside existing assessment approaches) This aligns with exploring potential prognostic capabilities and as an outcome measure for monitoring effectiveness of treatment

a Acknowledgement that bones/cartilages cannot directly be ‘viewed’ on US, only inferred.

US can support the likely differentials generated from the SLT clinical assessment, providing a foundation to move towards the use of US as a ‘rule in’ screening or assessment tool. For example, poor oral containment of a liquid bolus may be hypothesised from the SLT clinical swallowing assessment and further supported (or confirmed) with US when the soft tissue structures of the tongue-base and soft palate are not observed to contact during the oral phase of swallowing. This contrasts with a ‘rule out’ approach (more typically employed by imaging professionals such as radiologists and sonographers) where a range of potential sonographic findings (and subsequent clinical differentials) may be ruled out via the imaging. In the example above, US would/could not be used to ‘rule out’ other swallowing deficits, such as delayed initiation.

Describing US scope of practice for SLT clinicians also determines which imaging practises should not be performed. Examples of imaging not listed in Table 3 and therefore considered outside the SLT scope of practice might include

- space occupying lesions in the head and neck;
- vascular imaging of the head and neck;
- musculoskeletal or maxillofacial issues related to the head and neck, such as temporomandibular junction (TMJ) disorders;
- head and neck movements outside that of speech, voice and swallowing, such as blepharospasm and fasciculations.

While the above lie outside of SLT scope of practice, they may be identified as either incidental or concurrent imaging findings. Just as an SLT has a duty of care to escalate patient elements that may be strictly out of remit such as evidence of abuse or risk of self-harm, it is also necessary that they can act upon any unexpected imaging concerns.^15^ In this regard, a clear protocol must be in place for the clinician to be able to discuss concerns and for the clinical assessment and/or imaging of the patient to be escalated. A precedent for this has been established in other emerging SLT service models^16,17^ and could include lines of established communication with those who have more specialist US imaging expertise, access to other imaging modalities and/or surgical or medical opinion. The benefits of SLTs undertaking their US imaging as part of a wider clinical and/or imaging team become apparent in such situations.

The US report should be written and issued by the SLT undertaking the US activity and viewed as an integral part of the process or examination.^15^ Findings should be clearly and accurately communicated to the patient and other care pathway members either orally and/or via a written report. The format and nature of the reporting will vary according to the purpose for which US has been used; however, a formal written report is required where US has been applied for assessment or diagnostic purposes.^18,19^

## Education and competency

As per Figure 1, the education and competency elements must align with, and should be reflective of, the scope of practice. In this regard, a description of SLT-specific components is outside the remit of this paper, but would include both formal and informal training, supervision and support from an appropriately trained individual in this area, mentoring and feedback regarding pathology, clinical reasoning and clinical management.

A core consideration for any area of US is that while the scope of the scan may be limited, the standards must be the same as for imaging professionals such as radiographers and sonographers.^15^ Certified training courses specifically for SLTs do not currently exist; however, best practice guidance for the acquisition and maintenance of SLT competence can be developed via expert consensus, utilising occupational training standards^19–22^ and those developed for other professions as appropriate.^8^

A ‘foundation’ US curriculum is initially proposed in Table 4. Levels 1 (foundation), 2 (intermediate) and 3 (advanced) have been used to guide the level of skill required for each application (or scope of US practice) described in Table 2.

**Table 4.** Proposed curriculum for Speech and Language Therapists who wish to integrate ultrasound into their clinical practice. Educational elements have been divided into three components: (1) theoretical understanding, (2) technical skill and (3) analysis and interpretation.

Education elements	Level of ultrasound application		
	Foundation (level 1)	Intermediate (level 2)	Advanced (level 3)
**Theoretical understanding**			
**1. Understanding of how an ultrasound image is generated** Includes: • Fundamental physics as applied to ultrasound • Artefacts and how to manage / interpret them	Basic level of knowledge required; to include physics of ultrasound, echogenicity of tissues limited to those they are imaging.	Moderate-level knowledge required; allows operator to understand what grey-scale images of all speech/swallowing structure represent.	High-level knowledge required; includes extended US imaging such as Doppler or quantitative muscle US. Provides foundation for the operator to apply this core knowledge to assist in undertaking a differential diagnosis.
**2. Safety and professional considerations** Includes: • Thermal and non-thermal effects; ALARA principles • Awareness of limitations of ultrasound imaging and awareness of role of other imaging modalities • Infection control • Use of protocols; taking and labelling of standardised views • Reporting terminology; secure storage of images	Basic level of knowledge required due to limited scanning duration, and non-diagnostic, non-invasive role.	Moderate level of knowledge required; due to increased scanning duration and biofeedback role.	High-level knowledge required due to potential scanning duration; and diagnostic (potentially invasive) role.
**ii) Technical skill**			
**3. Image acquisition & optimisation** Includes: • The function of ultrasound machine settings (relating back to fundamental physics principles) • Knowledge of different types and purposes of ultrasound transducers • ‘Knobology’^a^ and application of image optimisations strategies in practical scenarios • Adaptation of imaging based on factors such as high BMI, poor patient positioning or anatomical variants.	Basic level of skill required to enable ‘plug and go’ application and simple adjustments to account for range of normal differences in size of head/neck anatomy. US is inaccessible if ‘fine tuning’ is required.	Moderate-level skill required; allows operator to ‘drive’ the machine to accurately identify and optimise the image for the target tissue(s).	High-level skill required; allows operator to ‘drive’ the machine to accurately identify a range of normal and pathological presentations in a range of tissue types. May include settings related to avoidance of neurovascular structures and accurate needle placement such as Spectral Doppler, Power Doppler, needle guidance/enhancement.
**Analysis & Interpretation**			
**4. Static imaging of speech, voice, and swallowing structures** Includes: • Ability to use standardised protocols, recognise normal structures and variation in anatomy.	Basic level of skill required, limited to just one structure of interest.	Moderate-level knowledge, skill and demonstrable competency required applied to a limited range of target tissue types.	High-level knowledge, skill and demonstrable competency required due to wide range of target tissue.
**5. Qualitative evaluation of speech, voice, and swallowing movement** Includes: • Ability to use standardised protocols, recognise normal vs abnormal variation in speech/swallowing movement.	Basic level of skill required, limited to just one set of movements and one function (speech vs swallowing) of a single structure (non-diagnostic).	Moderate level of skill required, limited to just one sets of movements and one function (speech vs swallowing) of more than one structure (non-diagnostic).	High-level knowledge required extended to more than one sets of movements and/or function (speech vs swallowing) in more than one structure (may be diagnostic).
**6. Quantitative analysis of speech, voice, and swallowing movement** Includes: Ability to use standardised protocols, measurement of speech/swallowing movements.	Not applicable.	Moderate level of skill required, limited to just one set of movements and one function (speech vs swallowing) of one or more structures (likely to be diagnostic).	High level of skill required, extended to more than one set of movements and/or function (speech vs swallowing) in more than one structures or modality (which may include quantitative muscle ultrasound or Doppler) (likely to be diagnostic).

a Ultrasound-specific term referring to the competency of the operator in determining and refining ultrasound settings for image acquisition.

Parts of the curriculum (e.g. foundation level) have potential to be integrated at undergraduate/pre-registration level, whereas the intermediate and advanced curriculum might align with the aspirational scope of practice as a diagnostic tool, prognostic indicator or outcome measure for monitoring effectiveness of treatment. Training at this level is likely to be undertaken by more experienced clinicians.

The level of education and training an SLT requires will be dictated predominantly by the requirements of their job role, rather than their banding or years of expertise. For example, injection of botulinum toxin into the salivary glands is likely to require advanced training as well as governance and recognition as an extended scope of practice, compared with the skills and competency required for providing biofeedback for speech sound disorders. The SLT must have the pre-requisite competencies required for their job role prior to integration of US as an education, treatment and/or assessment tool. Training programmes should include the principles and practicalities of ergonomic US practice as well as the safe use and potential hazards of diagnostic ultrasound equipment.^23^

### Advanced clinical practice agenda

As a progressive area of highly skilled practice, the use of US for assessment and diagnostic purposes would seem to naturally align with the advanced clinical practice agenda.^24^ We advocate though that US has the potential to become a routine part of SLT practice and that as such these clinicians do not *need* to be operating at ‘advanced level’ or above. Nonetheless, the four pillars of advanced practice (clinical practice, leadership and management, education and research) overlap substantially with the expanding role, that is, the use of US by SLTs.^24^ As such, we encourage US adopters to explore how use of the imaging modality can further advanced clinical practice and consultant roles.

## Insurance and governance

US is a non-regulated imaging modality; thus, no legal restrictions inhibit practice in this area. The use of US is recognised by RCSLT as an ‘extended scope’ of practice. As such, insurance is provided to its members provided the appropriate training and competency elements are in place; however, insurance is not an ‘exact science’ and each claim is usually assessed on its own merits. Activities that fall outside the remit of an SLT (e.g. use of US for muscle biopsy) require alternative cover and accountability agreed with the employer/provider.

Defining the scope of practice confers numerous governance and care pathway benefits. This includes awareness by other care pathway members of what the scan is and is not undertaken for, and support from clinical managers in care pathway design and staffing.

The use of terminology to explicitly clarify the nature of the scan is encouraged. An example of the professional context to the imaging process that could be communicated to colleagues is

Aligning with the scope of clinical and sonographic practice outlined for SLTs performing US (**this publication**), this scan is undertaken for the purposes of assessing/treating XXX as an adjunct to XXX as part of SLT management. The identification of other anatomical or pathological elements is explicitly beyond the scope of practice of the clinician. Therefore, the scan cannot be relied upon to either confirm or exclude any such anatomical or pathological elements.

Quality assurance considerations include data protection, storage of images/videos, testing of ultrasound devices^23^ as well as continuous professional development, and access to a second opinion. As US is often undertaken in non-radiology settings, direct access to picture archiving and communication system (PACS) for secure storage and backing up of sonographic images may not be available. This may pose a risk to data security as well as continuity of care and the ability to review image quality. Mechanisms for the secure storage of sonographic images/videos will need to be addressed in line with the information governance policy of the employer. Storage may include bespoke mechanisms to upload to PACS, or the use of other secure image storage capacity as advised by a data compliance officer. There are circumstances where recording of US data is often not required, for example, when used for professional training purposes or biofeedback therapy.

Peer review of the ultrasound images and reports should form part of the quality assurance process, particularly in the emerging areas of assessment and diagnostic practice. A peer-review audit tool for such purposes is offered by The British Medical Ultrasound Society (BMUS).^25^

## Broader considerations

### Expansion of scope of practice

Description of SLT clinical and sonographic scope of practice is not intended to stifle innovation or development of clinical practice or roles. Examples of expanded scope are provided in Table 2 and align with the advanced clinical practice agenda.^24^ Such activity may include the potential for SLTs to use US to make tracheal measurements for the purpose of tracheostomy insertion^26–28^ as well as confirmation of injection site of botulinum toxin in patients with sialorrhea.^29^ Applying the principles outlined in this paper means that where the activity demonstrably sits within the SLT management of a patient, then professional regulation and RCSLT insurance considerations would conceivably have already been addressed. Education and demonstrable competency considerations would need to be satisfied as well as any documentation required by the employer clinical governance committees that demonstrates the change in clinical practice is safe and regularly evaluated.

Another permutation might be where an SLT commences a parallel or advanced clinical activity which involves US imaging in a role that sits outside of what would otherwise be considered part of the SLT management of patients with speech or swallowing disorder. An example might include US-guided muscle biopsy of the muscles involved in speech and/or swallowing to support neurological diagnosis. RCSLT insurance considerations may not apply in such cases; therefore, a potential alternative route would be to arrange indemnity insurance via an employer. Again, education and demonstrable competency considerations would need to be satisfied along with agreement with clinical managers.

### Research

Given the sparsity of research evidence to support the application of US in the SLT profession, it is imperative to develop the evidence base relating to if, where and how US can enhance clinical effectiveness and efficiency of SLT assessment and treatment pathways. This includes consideration of optimal education and service delivery models as well as whether the use of imaging may have a negative impact on clinical outcomes or efficiency of resource use. The research priorities in this area are described in a recently published consensus paper.^5^

In relation to SLTs performing diagnostic US, some evidence, including a diagnostic test accuracy analysis in relation to the use of US to detect vocal fold palsy, can be drawn from other professional groups such as intensivists, anaesthetists and ear, nose and throat (ENT) medical practitioners.^30,31^ Nonetheless, the evidence base for the use of diagnostic US by SLTs needs development. The overlap with ENT practitioners, intensivists and head and neck sonographers provides potential opportunity for pooled research and inter-professional collaboration.

In addition to research which seeks to demonstrate the effectiveness of US biofeedback and evaluate assessment protocols, ongoing work seeks to improve the US technology. For speech assessment, systems are now available which synchronise the audio and US signals for play back and analysis.^32^ To analyse tongue shape and movement, the surface of the tongue must be tracked accurately. Ongoing work seeks to refine automatic tracking for both speech and swallowing assessment,^33–35^ allowing the analyst to extract numerical values to measure movement. Another approach involves machine learning to classify images. An example of this includes recent work to determine the correctness of articulatory gestures in children with speech disorders automatically,^36^ an approach which can also be used for outcome measurement. Further work using machine learning to classify various speech, swallow and laryngeal functions is likely in the future.

### A direction of travel for other specialities and geographical regions

This paper specifically reflects the situation for SLTs in the United Kingdom, and in this regard, it is noted that the level of autonomy is perhaps greater than that of some professionals in other countries. It is hoped therefore that the generic mechanisms outlined in this paper will provide a potential direction of travel for such professions and regions to advance their use of US imaging in a robust and sustainable manner.

## Conclusion

This paper presents a framework approach to support use of US in the SLT profession. As the uses of US in SLT are at different stages of development, scope of practice will depend on the specific area in which the SLT wishes to practice and the multidisciplinary support available to them. The combined (1) clinical usability and (2) clinical utility of US imaging according to the literature are key determinants of the scope of practice in this area. This encompasses a broad range of imaging elements relating to the assessment and therapeutic management of patients with speech, voice and/or swallowing disorders.

Education and competency assessment considerations are explicitly aligned with the clinical and sonographic scope of practice and provide the foundation for robustly satisfying a range of governance requirements. These are further addressed with elements such as data security and continuing professional development.

The framework provides an adaptable model for supporting expansion of US across a range of SLT specialities, including those outside of the current scope of SLT practice.
